# Survival Strategies and Metabolic Interactions between Ruminococcus gauvreauii and *Ruminococcoides bili*, Isolated from Human Bile

**DOI:** 10.1128/spectrum.02776-21

**Published:** 2022-07-11

**Authors:** Natalia Molinero, Elena Conti, Alan W. Walker, Abelardo Margolles, Sylvia H. Duncan, Susana Delgado

**Affiliations:** a Functionality and Ecology of Beneficial Microbes (MicroHealth) Group, Department of Microbiology and Biochemistry of Dairy Products, Dairy Research Institute of Asturias (IPLA)-Spanish National Research Council (CSIC), Villaviciosa-Asturias, Spain; b Instituto de Investigación Sanitaria del Principado de Asturias (ISPA), Oviedo-Asturias, Spain; c Gut Health Group, Rowett Institute, University of Aberdeen, Foresterhill, Aberdeen, Scotland; University of Minnesota

**Keywords:** syntrophy, survival, cross-feeding, co-cultures, ruminococci, bile resistance, SCFAs, stress conditions

## Abstract

Little is known about the bacteria that reside in the human gallbladder and the mechanisms that allow them to survive within this harsh environment. Here we describe interactions between two strains from a human bile sample, one Ruminococcus gauvreauii (IPLA60001), belonging to the *Lachnospiraceae* family, and the other, designated as Ruminococcoides bili (IPLA60002^T^; DSM 110008) most closely related to Ruminococcus bromii within the family Ruminococcaceae. We provide evidence for bile salt resistance and sporulation for these new strains. Both differed markedly in their carbohydrate metabolism. The R. bili strain mainly metabolized resistant starches to form formate, lactate and acetate. R. gauvreauii mainly metabolized sugar alcohols, including inositol and also utilized formate to generate acetate employing the Wood Ljungdahl pathway. Amino acid and vitamin biosynthesis genomic profiles also differed markedly between the two isolates, likely contributing to their synergistic interactions, as revealed by transcriptomic analysis of cocultures. Transcriptome analysis also revealed that R. gauvreauii IPLA60001 is able to grow using the end-products of starch metabolism formed by the R. bili strain such as formate, and potentially other compounds (such as ethanolamine and inositol) possibly provided by the autolytic behavior of R. bili.

**IMPORTANCE** Unique insights into metabolic interaction between two isolates; Ruminococcus gauvreauii IPLA60001 and Ruminococcoides bili IPLA60002, from the human gallbladder, are presented here. The R. bili strain metabolized resistant starches while R. gauvreauii failed to do so but grew well on sugar alcohols. Transcriptomic analysis of cocultures of these strains, provides new data on the physiology and ecology of two bacteria from human bile, with a particular focus on cross-feeding mechanisms. Both biliary strains displayed marked resistance to bile and possess many efflux transporters, potentially involved in bile export. However, they differ markedly in their amino acid catabolism and vitamin synthesis capabilities, a feature that is therefore likely to contribute to the strong synergistic interactions between these strains. This is therefore the first study that provides evidence for syntrophic metabolic cooperation between bacterial strains isolated from human bile.

## INTRODUCTION

The human gastrointestinal tract (GIT) is colonized by a variety of different microbial species that play vital roles in the maintenance and regulation of host homeostasis. Important mechanisms for host health include the metabolism of non-digestible dietary polysaccharides, and the production of vitamins and other metabolites with a beneficial effect, such as short-chain fatty acids (SCFAs), among others (1). The bacterial communities that inhabit our GIT have been widely characterized in the last few years, but little is known about the microbial communities present in the gallbladder and bile.

Recently, we characterized the human bile microbiome in subjects without any hepatobiliary pathologies (2). The bile of the gallbladder is colonized by a diverse microbiota, dominated by members of the Firmicutes, Bacteroidetes, Actinobacteria, and Proteobacteria phyla. During the course of this previous work, we were able to isolate, among others, two strains from a human bile sample, named IPLA60001 and IPLA60002, belonging to the *Clostridiales* order. The 16S rRNA gene sequences of these new strains showed the highest similarity with Ruminococcus gauvreauii (99%) and Ruminococcus bromii (93%), respectively.

At present, Ruminococcus is considered a polyphyletic genus, with species belonging to two Firmicutes families: Lachnospiraceae and Ruminococcaceae (3). There is therefore an urgent need to reclassify many of these bacteria into other genera in order to resolve the discrepancy of strains belonging to either the family Ruminococcaceae or Lachnospiraceae (3–6). Strain IPLA60002 showed phenotypic and genomic differences from the most closely related R. bromii strains, and it has now therefore been designated as a new species named Ruminococcoides bili, belonging to a new genus, Ruminococcoides, which is phylogenetically distant from other “true ruminococci” (7). R. bili belongs to the Ruminococcaceae family of the Firmicutes phylum and is a strictly anaerobic Gram-positive coccus. R. bili, like the most closely related R. bromii, is an efficient degrader of resistant starches, an important source of non-digestible dietary polysaccharides in the human colon (7, 8). The fermentation of these substrates provides nutrients for other bacteria and SCFAs, which are beneficial for host health (9). In contrast, R. gauvreauii strains are strictly anaerobic Gram-positive cocci, belonging to the Lachnospiraceae family of the Firmicutes phylum, and can use different sugar alcohols as carbon sources such as d-sorbitol, d-mannitol and inositol, producing acetate as their main fermentation product (10).

Microbial interactions in mixed communities are complex and can be mutualistic or antagonistic. Metabolite cross-feeding is a key feature of microbial ecosystems, but these likely complex interactions are generally not well characterized. Metabolite cross‐feeding can involve one strain utilizing, for example, macronutrient break down products, vitamins, or SCFAs formed by another strain. Cross‐feeding interactions can influence the metabolic pathways involved in fermentative metabolism and hence the energetics of bacterial metabolism. For example, formate, acetate and lactate can be formed by certain species, which are then used by other cross-feeding bacteria (11, 12). A previously demonstrated example is the interactions between R. bromii and Blautia hydrogenotrophica, as the latter can cross-feed on the formate generated by R. bromii while growing on resistant starch to generate acetate, employing the Wood‐Ljungdahl pathway (13). In our study, the R. gauvreauii IPLA60001 and R. bili IPLA60002 (DSM 110008) strains were isolated together and were difficult to cultivate separately, suggesting a potential symbiotic relationship. In this work, we investigated the trophic co-operation between these two human bile isolates using a combination of biochemical, genomic, and transcriptomic tools to analyze the production and cross-feeding of SCFAs and their metabolic interactions when cocultured on dietary starch as the sole carbon source.

## RESULTS

### Description of the strains and growth conditions.

R. gauvreauii IPLA60001 and R. bili IPLA60002 were isolated previously from a human bile sample (2). Growth conditions of R. bili IPLA60002 and its behavior on different media have been previously described (7). Both strains grow on Gifu Anaerobic Medium (GAM) plates supplemented with cysteine (GAMc), with R. gauvreauii IPLA60001 forming small white colonies after 72 h. In GAMc broth, R. gauvreauii required 18–20 h to reach the maximum OD_650_ (0.9–1.1). In M2GSC broth medium (14), supplemented with 30% clarified bovine rumen fluid (CBRF), the R. gauvreauii strain grew faster than in GAMc broth, reaching a maximum OD_650_ in 12 h, as was also reported previously for R. bili (7). As described previously, *R. bili* IPLA60002 has the ability to sporulate (7), and this behavior was also observed in R. gauvreauii strains IPLA60001 and DSM-19829 following staining using the Schaeffer and Fulton Spore Stain kit (Fig. S1 in the supplemental material).

### Molecular and phylogenetic identification.

The 16S rRNA genes from the strains R. gauvreauii IPLA60001 and R. bili IPLA60002 were amplified and sequenced. Molecular and phylogenetic description of R. bili IPLA60002, and its comparison to those of the related R. bromii (L2-63 and 5AMG) strains have been described previously (7). BLASTn analysis of strain IPLA60001 revealed that it shared 99% sequence similarity with R. gauvreauii. From whole genome sequence analysis of the IPLA60001 strain and following assembly, we obtained 60 contigs. The genome analysis of R. gauvreauii IPLA60001 showed a genome size of 4.14 Mb, a G+C percentage of 47.65%, the presence of 3,864 predicted open reading frames (ORFs) and 82 tRNAs. By comparing to the previously described genomic analysis of R. bili strain IPLA60002 (7), these results revealed markedly phylogenetic differences between the two bile isolates.

### Carbohydrate utilization and SCFA formation.

The carbon sources utilized by R. gauvreauii IPLA60001 were analyzed, comparing their fermentation capabilities with those of the related strain R. gauvreauii DSM-19829. The carbon sources utilized by R. bili IPLA60002 were analyzed previously (7) and compared their fermentation capabilities with those of the related strains; R. bromii L2-63 and 5AMG. Substrate utilization was the same for the two R. gauvreauii strains, with both able to use d-glucose, d-mannitol, d-sucrose, d-xylose, d-sorbitol, and inositol, and producing acetate as a major fermentation product. Regarding the *R. bili* IPLA60002 and the *R. bromii* strains, the ability to use a range of different carbon sources was also similar for all, resulting in the production of acetate and formate as major fermentation products (7). When comparing the different species, R. gauvreauii strains IPLA60001 and DSM-19829 are not able to hydrolyze resistant starches, in contrast with the R. bili and R. bromii strains tested previously (7).

**FIG 1:**
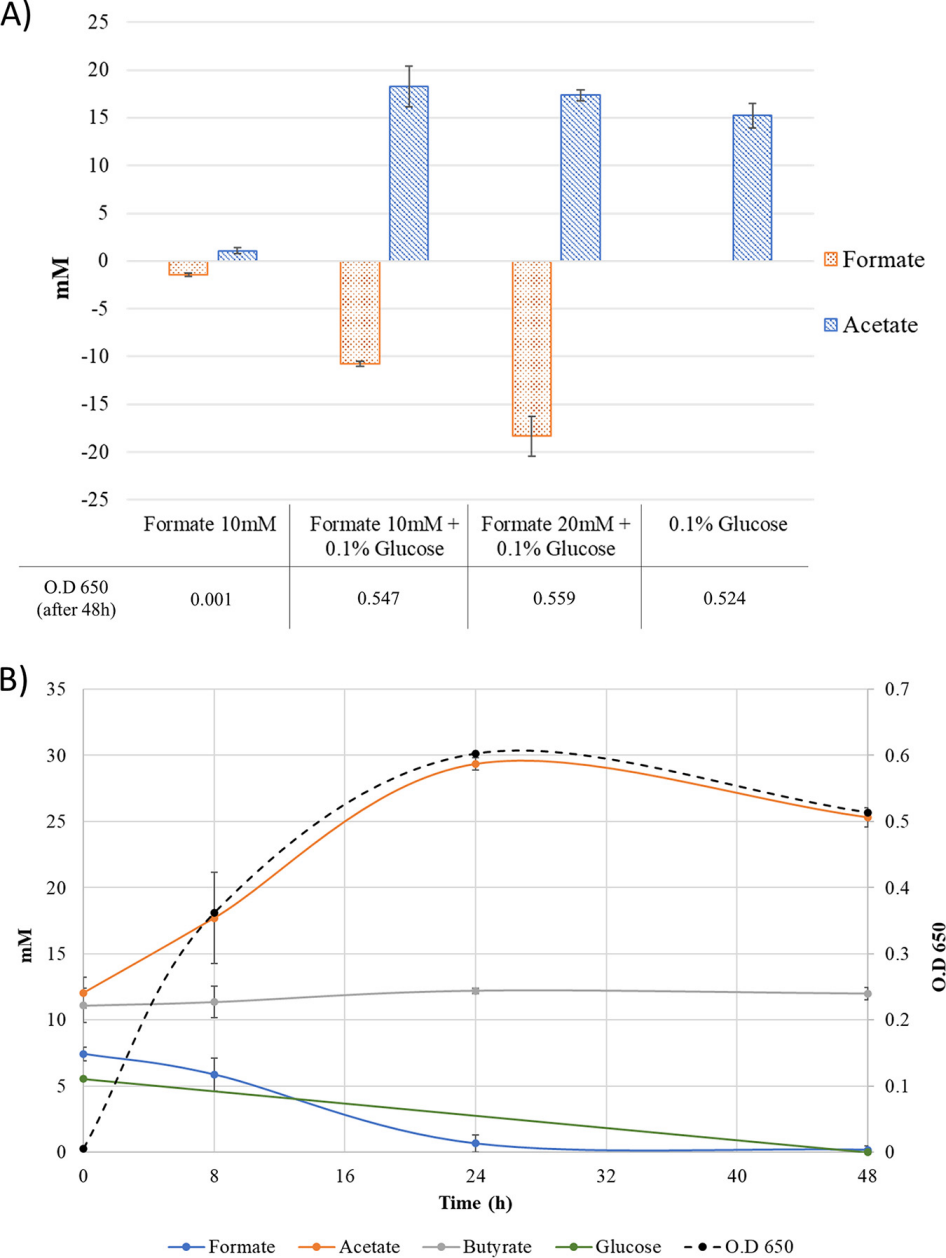
A: Short chain fatty acid profile of Ruminococcus gauvreauii IPLA60001 following 48 h growth on M2 medium supplemented with 30% clarified bovine rumen fluid, with different concentrations of formate and limited carbon source (0.1% glucose) and O.D. 650 values after 48 h. The results are normalized to time zero. B: Growth of Ruminococcus gauvreauii IPLA60001 on M2 medium supplemented with 30% clarified bovine rumen fluid, 10 mM formate, 10 mM butyrate and 0.1% glucose. The graph shows the variation in time (h) of short chain fatty acids and glucose concentrations (normalized to time zero) as well as the change in optical density (OD_650_).

As highlighted earlier, initial work with the new isolates showed that they grew very well in the presence of each other and were more difficult to cultivate separately. This raised the possibility that this may be driven by cross-feeding or syntrophy between the two species. Therefore, to test the hypothesis that R. gauvreauii IPLA60001 was able to use the formate produced by *R. bili* IPLA60002, the strain was grown in M2 medium supplemented with 30% CBRF and different concentrations of formate, alongside only limited additional carbon source (0.1% d-glucose). The CBRF also contains acetate, as can be seen in Fig. 1. After 48 h of culture, supernatants were analyzed by gas chromatography (GC), which showed that, although R. gauvreauii IPLA60001 was not able to grow with formate as the sole carbon source, when the medium was supplemented with glucose the strain completely metabolized all the formate added to the medium, at both 10 mM or 20 mM final concentrations (Fig. 1A). The SCFA profiles at different times of growth in cultures supplemented with formate, butyrate and limited carbon source showed that formate was metabolized within the first 24 h, during late exponential phase, when the strain was fermenting the glucose to produce acetate (Fig. 1B). In stationary phase and when the glucose was completely metabolized, the strain seemed to metabolize the acetate produced, although no changes in the SCFA profile were detected, so it does not appear that R. gauvreauii uses acetate to produce other SCFAs. Butyrate was not consumed or produced by R. gauvreauii IPLA60001, with levels constant from start to finish of the incubation, reflecting the initial concentration of butyrate in the CBRF added to the growth medium (Fig. 1B).

### Resistance to bile salts.

The resistance to bile salts was tested for all of the above-mentioned strains. Results for R. bili and R. bromii have been published previously and revealed a much higher bile tolerance for the bile isolate R. bili than the related colon-dwelling R. bromii strains (7), likely reflecting niche adaptation by R. bili to the gallbladder environment. The two R. gauvreauii strains showed the same strongly bile resistant phenotype as R. bili and were able to grow at the highest concentration tested (8%) of taurodeoxycholate (TDC), glycocholate (GC), taurocholate (TC), bovine bile, and a mixture of bile salts; at 0.1% of cholic acid (CA), and less than 0.25% of glycodeoxycholate (GDC) and porcine bile. This also likely reflects niche-adaptation by the R. gauvreauii IPLA60001 bile isolate to survival within the gallbladder.

### Comparative genomics of the bile isolates.

Genome analysis of R. gauvreauii IPLA60001 and R. bili IPLA60002 strains was performed using several different bioinformatic tools (see Materials and Methods). By comparing both genomes we found some differences related to bile salt stress response activities. The genome of *R. gauvreauii* IPLA60001 possesses genes related to activities against osmotic and oxidative stress, as well as some genes related to DNA repair that are not present in the genome of R. bili IPLA60002. In contrast, the genome of R. bili IPLA60002 possesses some genes related to stress response that are not present in the genome of R. gauvreauii IPLA60001, and some other genes related to DNA repair. R. bili IPLA60002 also possesses a choloylglycine hydrolase (EC 3.5.1.24) (7), an activity related to bile hydrolysis that is not present in the genome of the R. gauvreauii IPLA60001 strain.

**FIG 2:**
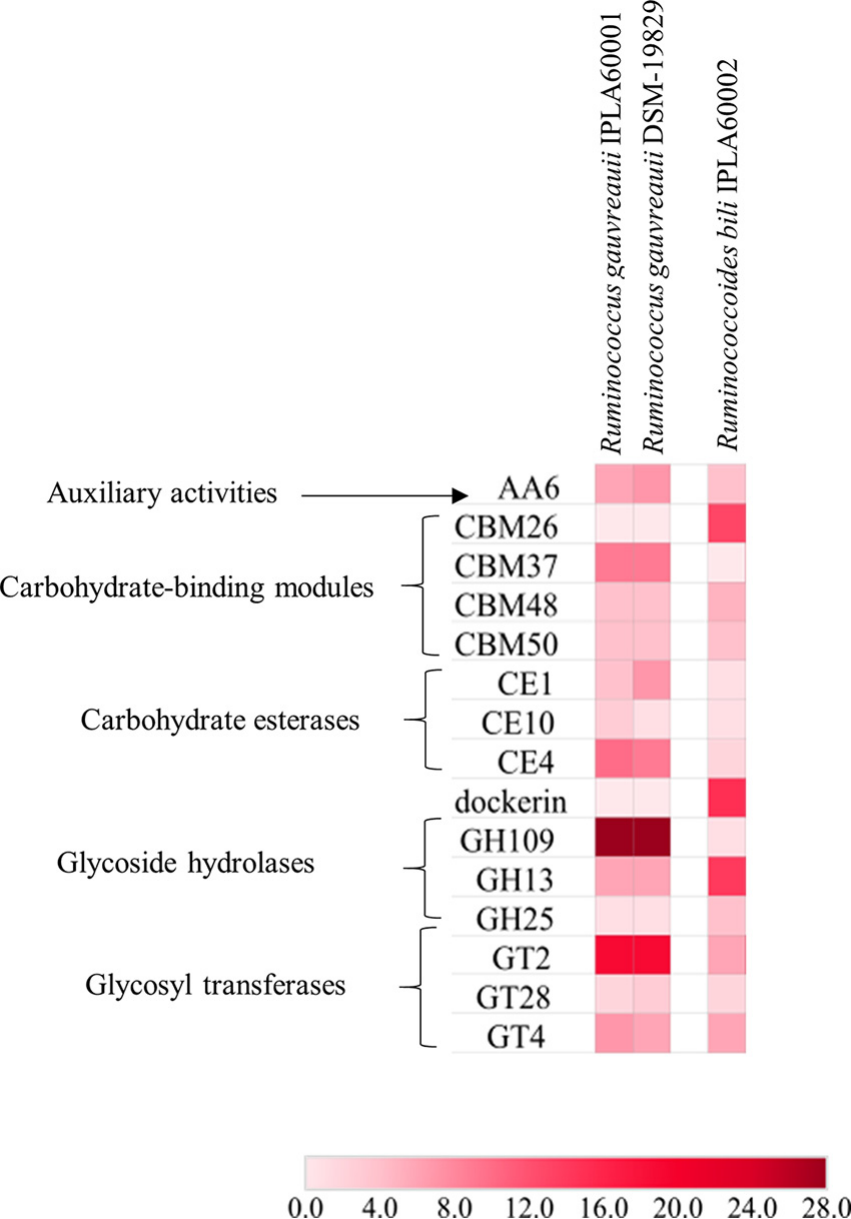
Heatmap based on dbCAN analysis comparing the functional carbohydrate active enzyme family classifications from CAZy in the genomes of Ruminococcus gauvreauii IPLA60001 *Ruminococcoides bili* IPLA60002 and Ruminococcus gauvreauii DSM-19829, and Ruminococcoides bili IPLA60002. Color key: each color represents a value range of presence of activities that was selected for optimal visualization ranging between 0 and 28 genes per genome.

Moreover, dbCAN analysis was used to provide information about the ORFs related to predicted carbohydrate-degradation enzymatic (CAZymes) activities. The genomes of the IPLA60001 and R. gauvreauii DSM-19829 strains were compared with those of *R. bili* IPLA60002 and its closely related strains of R. bromii, which were recently described in detail (7). The results of the dbCAN analysis are represented in a heatmap (Fig. 2) and revealed that the two R. gauvreauii strains had a similar CAZymes profile, with the presence of 28 genes related to the glycoside hydrolase family GH109 being particularly noteworthy. These genes corresponded with myo-inositol 2-dehydrogenases, which would explain the high inositol degradation rate observed for R. gauvreauii IPLA60001, which reached a maximum OD_650_ (0.9–1.1) in 8 h when grown in M2 medium supplemented with 0.2% inositol (data not shown).

The vitamin and amino acid biosynthesis and metabolism pathways of R. gauvreauii IPLA60001 and R. bili IPLA60002 were also analyzed, and these provided further evidence that there may be a mutually beneficial relationship between the two bile isolates (Table 1). Regarding vitamin biosynthesis, when comparing the genomes of the two strains, R. gauvreauii IPLA60001 has all genes needed to produce coenzyme B12 and cobalamin, which were not present in the R. bili IPLA60002 genome. Conversely, the R. bili IPLA60002 strain possesses all genes needed to produce coenzyme A, which are absent in the R. gauvreauii IPLA60001 genome. In relation to the amino acids metabolism, the results showed that R. gauvreauii IPLA60001 possesses all genes for putrescine utilization, urea decomposition, methionine and lysine biosynthesis, and creatine and creatinine degradation, none of which were detected in the R. bili IPLA60002 genome. Moreover, the latter possessed genes for glycine and serine utilization, and these pathways were not present in the R. gauvreauii genome. The genes related to these metabolic and biosynthetic pathways that were detected in at least one of the strains are represented in Table S1 in the supplemental material.

**TABLE 1:** Differences in the presence of amino acid and vitamin pathways between the genomes of Ruminococcoides bili IPLA60002 and Ruminococcus gauvreauii IPLA60001

Pathway	*R. gauvreauii* IPLA60001^*a*^	*R. bili* IPLA60002
Vitamin		
Coenzyme A biosynthesis cluster	−	+
Coenzyme B12 biosynthesis	+	−
Molybdenum cofactor biosynthesis	+	−
Lipoic acid metabolism	+	−
Cobalamin synthesis	+	−
Amino acid		
Putrescine utilization pathways	+	−
Urease subunits	+	−
Urea decomposition	+	−
Methionine biosynthesis	+	−
Lysine biosynthesis DAP pathway, GJO scratch	+	−
Creatine and creatinine degradation	+	−
Glycine and serine utilization	−	+

a -, genes for this pathway not detected; +, the strain has all genes required to produce this vitamin or amino acid.

### Interactions of R. bili IPLA60002 and R. gauvreauii IPLA60001 in coculture.

Co-cultures containing both bile-derived isolates were performed in order to investigate their potential to cross-feed. After 6 and 8 h of bacterial growth in coculture in M2 medium with rice starch (RS) as carbon source, transcriptomic analysis was performed. As expected, R. gauvreauii IPLA60001 was not able to grow in monoculture on RS as the sole added carbon source. Evidence showing growth of this strain in coculture with R. bili IPLA60002 would therefore indicate that IPLA60002 was producing breakdown products or metabolites from RS that R. gauvreauii IPLA60001 was able to grow on. The results obtained using RNA-seq (see below) confirmed that IPLA60001 was indeed growing in coculture with IPLA60002. To corroborate the ability of the R. gauvreauii strain to metabolize formate (see Fig. 1A), in this case produced by the R. bili strain in coculture, the SCFAs profiles of the cocultures and single cultures were tested for the same period of time. These results did not show the complete or partial formate consumption previously observed for R. gauvreauii IPLA60001in single culture, at neither the 6 h or 8 h time points (Fig. S2), probably because in these cases the time of sampling was earlier in the exponential phase and the consumption of formate likely occurs after 8 h of incubation, once cells enter stationary phase (see Fig. 1B), or because there could have been slight consumption of formate by R. gauvreauii that was masked by concomitant production by R. bili, thereby not resulting in appreciable differences in measured formate levels.

### Transcriptome analysis and changes in expression profiles.

**FIG 3:**
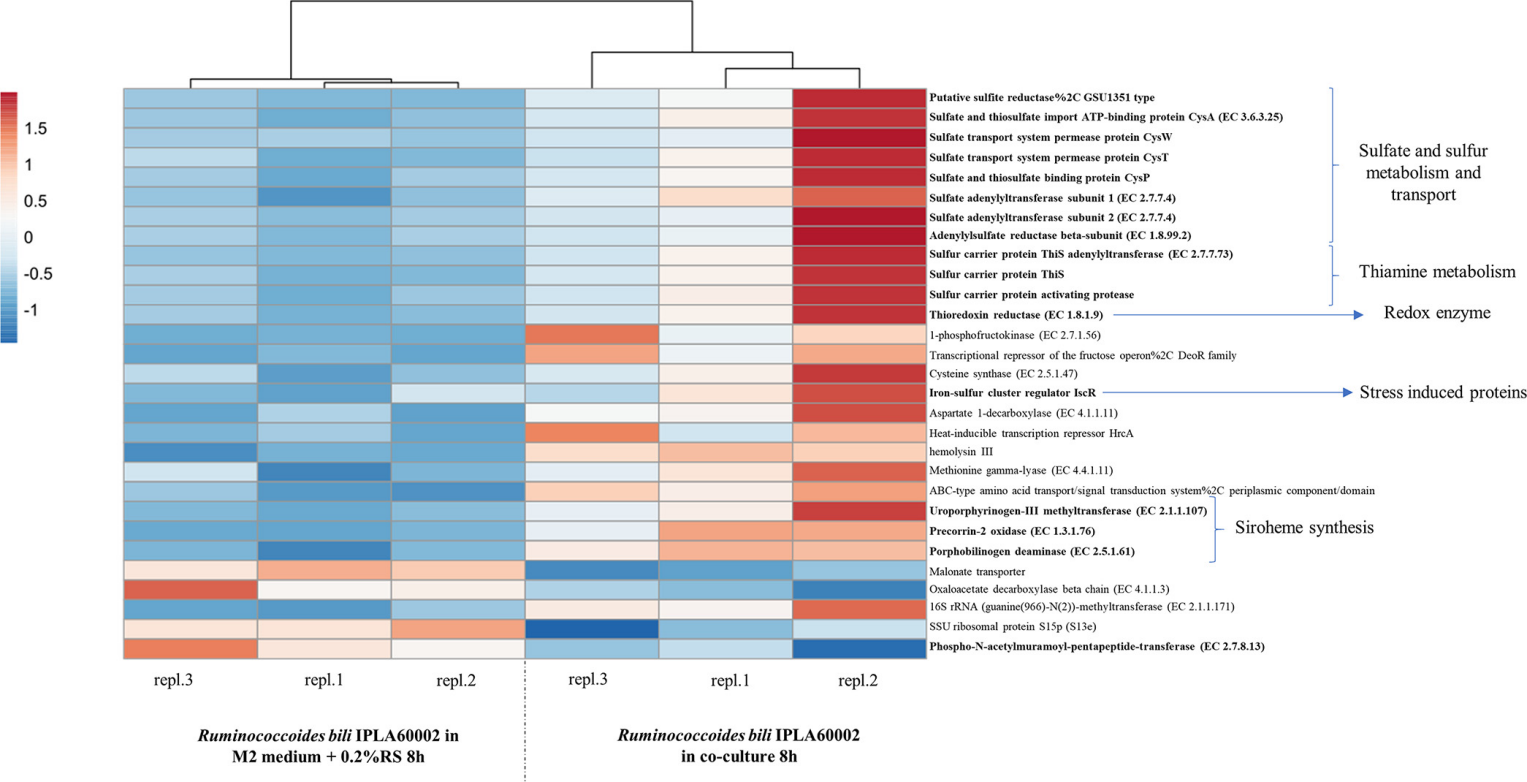
Heatmap of the transcription level of differentially expressed Ruminococcoides bili IPLA60002 genes when the strain was growing in pure culture or in coculture with Ruminococcus gauvreauii IPLA60001 for 8 h. The heatmap shows only the genes that were differentially expressed with a log_2_ (fold change) < -1 or > 1 and an adjusted *P*-value of 0.05. RS: rice starch.

To further investigate the cross-feeding relationship between R. gauvreauii IPLA60001 and R. bili IPLA60002, RNA-seq was used to analyze the transcriptome profile of each strain when grown alone and in cocultures. We performed the following comparisons: changes in the transcriptome of the R. bili strain when grown in mono- or in coculture (with R. gauvreauii IPLA60001) for 8 h; changes in the transcriptome of the R. bili strain between the 6 and 8 h time points of coculture (with R. gauvreauii IPLA60001); and changes in the transcriptome of the R. gauvreauii strain between 6 and 8 h time points of coculture (with R. bili IPLA60002). Transcriptomic analysis revealed that when R. bili IPLA60002 was in coculture with R. gauvreauii IPLA60001, certain R. bili IPLA60002 genes were upregulated. This included several genes related to sulfate and sulfur metabolism and transport, genes related to thiamine production, and genes related to siroheme synthesis (Fig. 3), as well as thioredoxin reductase and other genes encoding stress-induced proteins when R. bili IPLA60002 was in coculture. On the other hand, results showed a downregulation of genes related to malonate transport, oxaloacetate metabolism and peptidoglycan biosynthesis (Fig. 3), and the latter may be linked to autolysis of R. bili IPLA60002 at 8 h.

**FIG 4:**
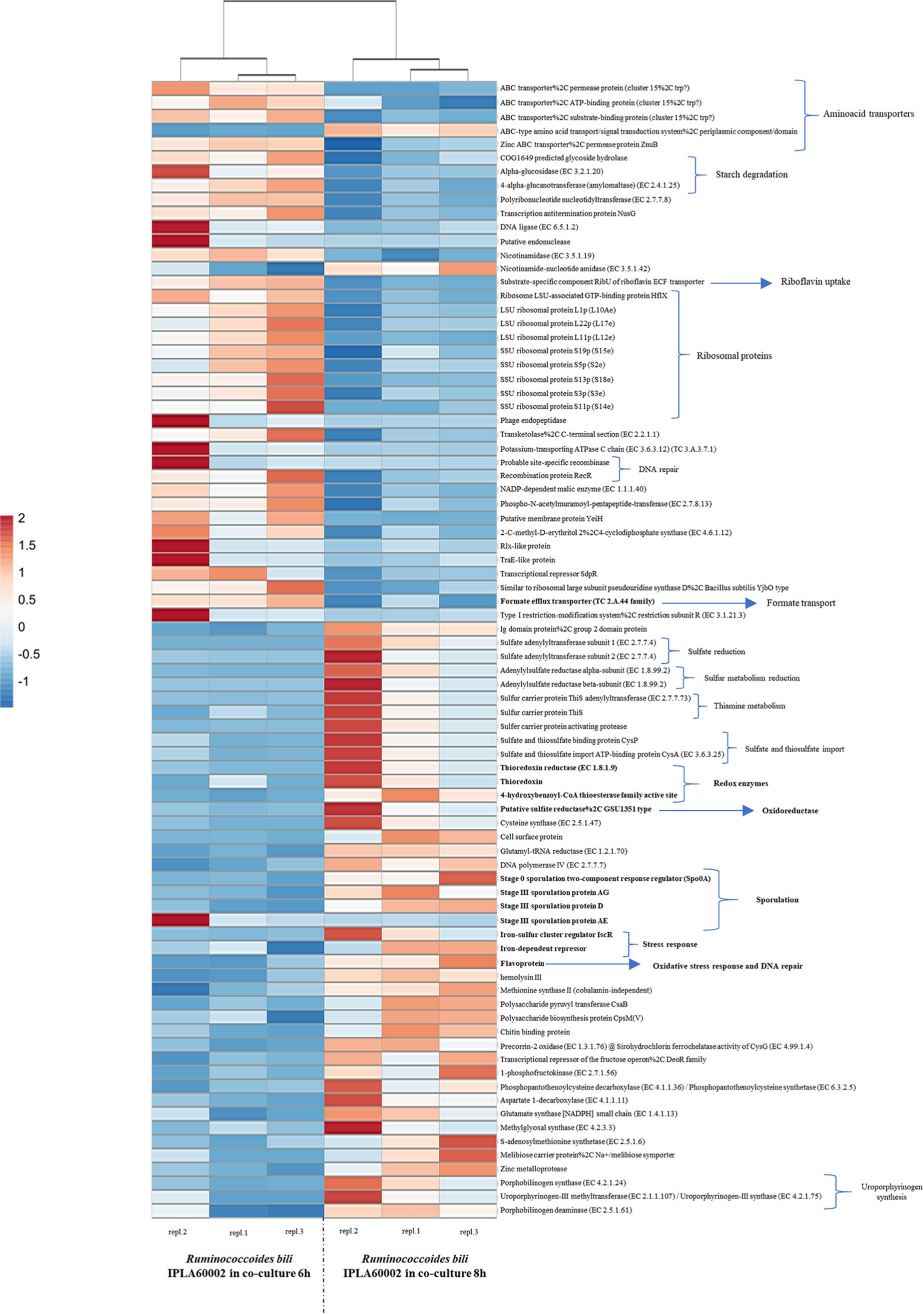
Heatmap of the transcription level of differentially expressed Ruminococcoides bili IPLA60002 genes between 6 and 8 h of growth in coculture with R. gauvreauii IPLA60001. The heatmap shows only the genes that were differentially expressed with a log_2_ (fold change) < -1 or >1 and an adjusted *P*-value of 0.05.

The most important changes in the transcriptional profiles of the R. bili strain IPLA60002 were detected when comparing the data from the 6 and 8 h coculture time points. At 6 h, genes related to starch degradation, several ABC transporters, ribosomal proteins and riboflavin uptake were upregulated, among others (Fig. 4). At 8 h, there was upregulation of genes related to stress response, highlighting activities related to sulfate reduction, thiamine production, and some redox enzymes, such as a thioredoxin reductase and one 4-hydroxybenzoyl-CoA thioesterase. Upregulation of an oxidoreductase enzyme was also detected, along with other stress response and DNA repair proteins, such as flavoproteins. In this comparison, genes for sporulation were also upregulated at 8 h. This is in agreement with previous work that confirmed the sporulation capability of R. bili IPLA60002 (7). Conversely, several genes related to replication and translation were downregulated in coculture at 8 h, highlighting amino acid transporters, ribosomal proteins, and DNA repair enzymes, which could be related to the possible autolysis of R. bili IPLA60002 from this time point.

The third comparison performed was to analyze changes in the transcriptome of R. gauvreauii IPLA60001 between 6 and 8 h of growth when in coculture with R. bili IPLA60002. In this case, at 8 h, ribosomal proteins were mainly upregulated, along with other genes related to replication, transcription and transduction (Fig. S3 in the supplemental material), suggesting a change in the growth rate of R. gauvreauii IPLA60001, which may have been increasing. We also observed upregulation of one gene related to ethanolamine utilization [acetaldehyde dehydrogenase (EC 1.2.1.10)].

The changes in the transcriptome of R. gauvreauii when the strain was grown in pure culture or in coculture with *R. bili* IPLA60002 for 8 h were also analyzed. In this case the mono- culture of R. gauvreauii IPLA60001 was performed in glucose (0.2%) since it does not grow on RS. Nonetheless, even considering this limitation, the comparison of the transcriptome showed that several genes for ethanolamine and inositol metabolism were upregulated when R. gauvreauii IPLA60001 was in coculture, such as ethanolamine utilization proteins EutA, EutJ and EutQ; one myo-inositol 2-dehydrogenase (EC 1.1.1.18) and one formate dehydrogenase (Table 2). The findings on formate metabolism corroborate the hypothesis that IPLA60001 is able to metabolize formate produced by R. bili IPLA60002.

**TABLE 2:** Transcriptional changes in Ruminococcus gauvreauii IPLA60001 after growth for 8 h alone or in coculture with *Ruminococcoides bili* IPLA60002^*a*^

Gene	log_2_ fold change	*p*-value	*p* adjusted
Acetaldehyde dehydrogenase (EC 1.2.1.10) ethanolamine utilization cluster	5.369	2.63E-31	8.86E-29
ATP:Cob(I)alamin adenosyltransferase (EC 2.5.1.17) ethanolamine utilization cluster	7.444	1.44E-24	2.42E-22
Ethanolamine ammonia-lyase heavy chain (EC 4.3.1.7)	3.948	5.54E-18	6.22E-16
Ethanolamine ammonia-lyase light chain (EC 4.3.1.7)	2.8383	3.77E-07	1.20E-05
Ethanolamine utilization polyhedral-body-like protein EutL	2.986	1.62E-07	5.68E-06
Ethanolamine utilization protein EutA	3.761	1.97E-08	8.52E-07
Ethanolamine utilization protein EutJ	6.966	4.03E-28	8.48E-26
Ethanolamine utilization protein EutQ	3.123	3.73E-10	2.17E-08
Ethanolamine utilization protein similar to PduT	7.686	2.81E-17	2.95E-15
Ethanolamine utilization protein similar to PduV	3.218	8.42E-06	0.00019
EutN-like protein clustered with choline trimethylamine-lyase	6.89	1.49E-18	1.80E-16
Formate dehydrogenase-O%2C major subunit (EC 1.2.1.2)	2.514	1.14E-10	7.36E-09
Myo-inositol 2-dehydrogenase (EC 1.1.1.18)	0.737	0.00935	0.05546
Sorbitol dehydrogenase	2.053	7.12E-05	0.00122

a The upregulated genes related to carbohydrate metabolism are represented.

## DISCUSSION

In this paper, behavior and interactions between two biliary-derived strains were characterized through phenotypic, genomic, and transcriptomic analyses. The strains were initially recovered by culturing together on the same agar plate from the same human bile sample, and subsequently proved difficult to isolate independently compared to the improved growth observed when cultured together. These observations indicated that there may be an inter-dependent relationship between these strains, and putative mutualistic interactions including cross-feeding were therefore evaluated.

Resistance to several bile salts was shown for both of the strains that were isolated from bile, which is in contrast with the most phylogenetically related species of fecal origin. In general, the two bile isolates showed more resistance to the primary conjugated bile salts, such as GC and TC, which are more common in the gallbladder, as well as to bovine bile and mixed bile salts mainly composed of TC, TDC, and GC. The phenotypic results observed for this type of resistance were supported by the genomic analysis, in which some genes potentially related to bile salt resistance and stress response were found. Both biliary strains possessed many genes annotated as different efflux transporters, such as ABC-type multidrug transporters and small multidrug resistance transporters that protect bacteria from an array of hydrophobic and cationic antimicrobials (15), as well as multidrug and toxic compound extrusion efflux family proteins, which have been related to the extrusion of cholate and deoxycholate in Escherichia coli (16). This repertoire of efflux transporters detected in the two bile isolates could therefore play a role in exporting bile salts. Genomic analysis also revealed the presence of a cholylglycine hydrolase (17) in the genome of R. bili IPLA60002 (7), an enzyme associated with the hydrolysis of bile salts that may be involved in their detoxification by this strain. The presence of these genes, together with those for sporulation (7) may sustain their survival in a hostile environment like the biliary tract. We confirmed the sporulation capability of both R. gauvreauii strains microscopically following staining with Schaeffer and Fulton Spore Stain, as was also previously observed in R. bili IPLA60002 (7). This ability to sporulate was also previously reported for the most closely related species to R. bili, R. bromii (18).

Regarding growth and fermentation capabilities, metabolic interactions between the two biliary strains were indicated by their individual behavior and fermentation abilities in monocultures, as well as demonstrated by genomic analyses and differential transcriptional responses in cocultures. We previously observed that major fermentation products from R. bili IPLA60002, which was able to grow in resistant starch, were acetate and formate (7). This latter compound is a key metabolite in the energy metabolism of some cross-feeding anaerobes (19). Both formate and hydrogen can be used for interspecies electron transfer (20), and at least in the species R. bromii the production of acetate and formate is accompanied with ethanol and hydrogen production (18). Products released from the metabolism of resistant starches by R. bromii, such as different reducing sugars including glucose and malto-oligosaccharides, can be used by other gut bacteria (8). In this work the R. gauvreauii IPLA60001 strain was not able to grow in formate as the sole carbon source. However, it metabolized formate during late exponential phase (over 8 h of incubation time), an observation that was in line with the results obtained by SCFAs and RNA-seq analyses at 8 h in cocultures on starch by the R. bili strain, suggesting that R. gauvreauii preferentially uses the products released from RS by R. bili for growth and then switches to metabolize the formate produced by R. bili once in late exponential phase. The potential for anaerobic bacteria to grow by converting formate to other compounds such as hydrogen and bicarbonate has been overlooked for years (21), but there are some existing reports about metabolic interactions and degradation of formate, mainly by methanogens (22). The study by Dolfing et al. reported that, after the addition of formate as the sole energy and carbon source, biomass only increased when strains were grown in cocultures (22). R. gauvreauii IPLA60001 is not able to grow in complex substrates such as starch, so when the strain grows in coculture with R. bili IPLA60002 the carbon sources available are the end products of the metabolism and hydrolysis of the starch. It is possible that upon starch degradation R. bili IPLA60002 needs at least 24 h to produce enough formate to be metabolized by IPLA60001, and plausibly during this time R. gauvreauii IPLA60001 was metabolizing other secondary products derived from starch metabolism by IPLA60002, as the utilization of formate was only observed during late exponential phase in pure culture (Fig. 1B).

When comparing the R. bili IPLA60002 transcriptome between 6 and 8 h of co-cultivation, gene expression changed from starch degradation and riboflavin uptake to sporulation and stress response expression at 8 h, which is consistent with the autolytic phenotype observed for this strain after 12 h in pure culture (7). Co-cultures of both strains might accelerate autolysis, even if both strains do not compete for the same substrates, because the autolysis phenomenon in bacteria is influenced by a variety of different factors including pH, growth phase or teichoic acids among others (23). In line with this, differential gene expression was detected when comparing the R. bili IPLA60002 transcriptome at 8 h alone or in co-cultivation, indicating that, at this time of growth, genes related to stress and sulfur metabolism were activated in the presence of R. gauvreauii IPLA60001. Additionally, the autolysis behavior of the R. bili IPLA60002 strain might release membrane phospholipids such as phosphatidylethanolamine and phosphatidylinositol, which would be in concordance with the ethanolamine and inositol metabolism upregulation observed in the strain of R. gauvreauii IPLA60001 in co-cultivation with R. bili IPLA60002, which could be providing favorable conditions to increase growth of R. gauvreauii IPLA60001.

Genome analysis of both strains, revealed differences in genes for vitamin, cofactor and amino acid production pathways, supporting the proposed syntrophic metabolic interactions and benefits between R. gauvreauii IPLA60001and R. bili IPLA60002. Of particular note was the exclusive presence of coenzyme B12 and cobalamin producing enzymes, essential for bacterial growth, as well as putrescine utilization enzymes in the genome of R. gauvreauii IPLA60001. Putrescine is a biogenic amine normally present in the CBRF fluid, and its degradation is common in strictly anaerobic bacteria, leading to the production of mainly acetate, butyrate, molecular hydrogen and ammonia (24). In this case, the degradation of putrescine by the R. gauvreauii strain could provide a nitrogen/carbon source to R. bili, which in return could provide, for example, Coenzyme A to R. gauvreauii, supporting the syntrophic interaction.

Our results reveal potential synergistic mechanisms between two microorganisms isolated from bile, which are of particular ecological interest in this scarcely studied niche. This work therefore represents an important step forward in the understanding of the environmental distribution and relatedness of symbionts in the human gallbladder. These species could play roles in the maintenance of the balance between the bile microbiome and host, and so impact on host physiology and health status (2, 25, 26). Our findings suggest the need for further future efforts to explore microbial syntrophic interactions from other parts of the human body, and to characterize novel bile-related functions that could be unique for bacterial members inhabiting this particular ecosystem.

## MATERIALS AND METHODS

### Isolation procedure for bacteria from human bile.

Human bile samples from the gallbladder of liver donors without any hepatobiliary disorder were obtained under aseptically controlled conditions during liver transplant at the General Surgery Service of HUCA (Central University Hospital of Asturias) in Spain during a previously described study (2). Bile samples were immediately transported refrigerated to the laboratory and cultivated in different media, including GAM (Nissui, Tokyo, Japan) supplemented with 0.25% (wt/vol) l-cysteine (Sigma-Aldrich, Saint Louis, MO) (named GAMc). The strains reported here, named IPLA60001 and IPLA60002, were isolated from a 2% agar GAMc plate incubated at 37°C for 72 h in a Whitley MG500 anaerobic cabinet (Don Whitley Scientific, West Yorkshire, UK) under a 10% H_2_, 10% CO_2_, and 80% N_2_ gas atmosphere. The strains were routinely maintained by growing in GAMc agar plates under anaerobic conditions. Other media, such as the semi-defined RUM medium (27), PYG (DSMZ Medium 104) and the M2GSC medium supplemented with 30% vol/vol CBRF, were also utilized during characterization of isolates.

### Bacterial strain and growth conditions.

Phenotypic analyses of IPLA60001 and IPLA60002 strains were carried out in parallel with the type of strain R. gauvreauii DSM-19829, purchased from the DSMZ Collection. If not otherwise specified, all strains were routinely maintained by growing anaerobically at 37°C for 16–18 h in M2GSC broth medium supplemented with 30% CBRF in Hungate tubes. Media were prepared anaerobically under a stream of 100% CO_2_.

### Identification of the isolates by 16S rRNA gene sequencing.

Total genomic DNA was extracted from 18 h grown cultures on GAMc broth using the GenElute Bacterial Genomic DNA kit (Sigma-Aldrich). Purified DNA was used as a template for amplification of the 16S rRNA gene using the universal primer pair 27 (forward primer) and 1492 (reverse primer) of the E. coli numbering system (28). PCR amplification products were sequenced using an ABI 373 DNA sequencer (Applied Biosystems, Carlsbad, CA) at the facilities of Macrogen (Madrid, Spain). The 16S rRNA gene sequences were compared with those deposited in the GenBank database using BLAST (https://blast.ncbi.nlm.nih.gov/Blast.cgi).

### Whole genome sequencing.

Genomic DNA from both biliary strains was purified using the “DNeasy blood and tissue” kit (Qiagen, Germany) following the manufacturer’s protocol. Genome sequencing was carried out using 250–290 paired-end libraries on an Illumina MiSeq Sequencing System (Illumina) at GenProbio SRL (Parma, Italy). Genome assembly was performed with the MIRA assembler v4.0.2 (29). ORFs prediction was performed with Prodigal v2.6 (30). Automatic annotation of the ORFs was performed with BLAST against the NCBI database and HMMER software against the PFAM database (31). The quality of the final contigs was improved using the Burrows-Wheeler Aligner (32), SAMtools suite (33) and VarScan v2.2.3 (34) software packages.

### Genome comparisons.

We compared the genomes of the two biliary strains IPLA60001 and IPLA60002 with that of R. gauvreauii DSM-19829. To perform the comparison between genomes the software RAST (Rapid Annotation using Subsystem Technology) was used, choosing the classic RAST tool as the gene annotation scheme. After gene annotation, differences in vitamin and amino acid synthesis pathway related genes were analyzed manually using the outputs from RAST. We also used dbCAN (35), a web server and database for genes encoding carbohydrate-active enzyme activities, to compare the different CAZymes profiles in each studied genome. All data in dbCAN are generated based on the family classification from CAZy website (http://www.cazy.org/).

### Bile salt resistance.

The analysis of resistance to different bile salts was performed comparing the two human gallbladder bile isolates IPLA60001 and IPLA60002 with the R. gauvreauii type strain DSM-19829. Comparisons were also made with R. bromii strains from the Rowett Institute (L2-63 and 5AMG), the results of which have been described previously (7). The analysis was carried out in triplicate by determining the MICs on M2GSC 2% agar plates supplemented with 30% CBRF and different concentrations (ranging from 0% to 12% wt/vol) of CA, TDC GC, TC, GDC, Porcine Bile Extract, Bovine Bile (purchased from Sigma-Aldrich), and Mixed Bile Salts (Oxoid, Waltham, MA). The plates were incubated at 37°C in the anaerobic cabinet for 48 h.

### Carbohydrate utilization and enzymatic profiles.

To analyze the growth of IPLA60001 and IPLA60002 in comparison with R. gauvreauii DSM-19829, we proceeded as previously carried out with R. bromii 5AMG and R. bromii L2-63 strains (7). We performed a series of characterization tests in M2 medium supplemented with 30% CBRF and 0.2% wt/vol of different carbon sources and resistant starches, in Hungate tubes (14). Triplicate tubes were inoculated with an overnight culture of the corresponding strain, and the OD_650_ values were recorded every 2 h for a period of up to 48 h in a Novaspec II Spectrophotometer (Amersham Pharmacia Biotech Inc., NJ). The growth in different resistant starches was recorded by measuring the drop in the pH (pH <6.0 as positive growth) after 48 h of incubation.

These analyses were completed using the API20A test system, which contains substrates for carbohydrate utilization and enzymatic reactions, according to the manufacturer’s instructions (bioMérieux, Marcy l'Etoile, France). The inoculated test strips were incubated anaerobically in the anaerobic cabinet at 37°C for 24 h.

### Sporulation ability of R. gauvreauii strains.

The ability of R. gauvreauii IPLA60001 and DSM-19829 strains to sporulate was assessed by growing the cultures for 72 h in M2GSC medium supplemented with 30% CBRF, followed by heating at 80°C for 20 min to kill vegetative cells. Subsequently, the cells were treated as described previously (18) with the Schaeffer and Fulton Spore Stain kit (Sigma-Aldrich) used to visualize spores by microscopy.

### Determination of SCFAs.

SCFA concentrations and acid production by the IPLA60001 and IPLA60002 strains were measured by gas chromatography as described previously (36) after growing the strains in M2GSC medium supplemented with 30% CBRF. Following derivatization of the samples using N-tert-butyldimethylsilyl-N-methyltrifluoroacetamide (Sigma-Aldrich), the samples were analyzed, using helium as the carrier gas, in a Hewlett Packard gas chromatograph system fitted with a silica capillary column linked to a Shimadzu chromatopac integrator (Dyson Instruments Ltd, Tyne and Wear, UK). Additionally, we studied the ability to metabolize formate and butyrate by *R. gauvreauii* IPLA60001. The strain was grown for 48 h in M2 medium supplemented with 30% CBRF and 0.1% glucose with different concentrations of formate (10 and 20 mM) and butyrate (10 mM).

Finally, the SCFA profiles of cocultures of IPLA60001 and IPLA60002 strains were monitored. The supernatants of the cocultures of the two strains in M2 medium supplemented with 30% CBRF and 0.2% rice starch at 6 and 8 h of growth were taken in triplicate, and the SCFAs analyzed as described above.

### Co-culture conditions.

To study the interactions between R. bili IPLA60002 and R. gauvreauii IPLA60001 we carried out coculture combinations in M2 medium with 0.2% rice starch (Sigma-Aldrich) as sole carbon source. The coculture conditions were as follows: 10 mL of initial exponential phase cocultures of IPLA60002 and IPLA60001 (6 h, OD_650_ 0.4–0.5), and 10 mL cocultures in middle exponential phase (8 h, OD_650_ 0.6–0.7). As controls, the strains were grown concurrently in monoculture. Three technical replicates were performed for each condition, and cultures were inoculated from stock liquid cultures at 1% of total liquid volume. The tubes were centrifuged for 10 min at 10,000 rpm and the pellet was immediately resuspended in 1 mL of RNAlater (Qiagen) and frozen at −80°C.

### RNA extraction and RNA sequencing.

Total RNA was extracted from the pellets that were resuspended in 200 μL of lysozyme (20 mg/mL) and mutanolysin (40 U/mL) solution and incubated at 37°C for 1 h. Then, 800 μL of QIAzol (Qiagen) was added and placed in a tube containing 0.8 g of glass beads (106 μm diameter, Sigma). The cells were lyzed by shaking the mix on a BioSpec homogenizer (BioSpec Products, Inc.) for 2 min followed by 2 min on ice. This step was repeated three times. Samples were further treated with 1 volume of chloroform, mixed and then centrifuged at 12,000 rpm for 15 min at 4°C. The upper phase containing the RNA was recovered. Samples were further purified using the RNeasy minikit (Qiagen) following the manufacturer’s protocol. Quality and integrity of the RNA was checked using an Agilent 2200 Tape Station Nucleic Acid System (Agilent Technologies, Palo Alto, CA). The amount of RNA was quantified using a Qubit 4 fluorometer with Qubit RNA assay kits (Thermo Fisher Scientific, Inc., Waltham, MA). One to 2.5 μg of total RNA was depleted using Ribo-Zero rRNA removal kit (Illumina) to remove rRNA according to the supplier’s instructions. The yield of rRNA depletion was checked using an Agilent Tape Station and the rRNA-depleted RNA samples were treated with the TruSeq Stranded mRNA sample preparation kit (Illumina). Size evaluation and quantification of RNA samples was performed as described above. The whole transcriptome library was sequenced using a NextSeq 550 Sequencing System (Illumina) with a High Output kit v2.5 (75 cycles) flow cell.

### Transcriptome analysis.

High quality reads for each sample were aligned against the reference genomes of IPLA60002 (NCBI accession number SRR9209608) and IPLA60001 (SRR10273262). The corresponding associated annotation files in *.gff* format were used to obtain information for downstream analysis. Alignments were generated using bowtie2 v.2.2.6 (37), and SAMtools v.1.4 (31) was used to get the *.bam* files and sort them into new files suitable for the next step. Read counts were generated using FeatureCounts (38). Gene expression for each sample was computed as a measure of the total number of reads uniquely aligning to the reference, binned by genic coordinates. Differential gene expression analysis was performed using the Bioconductor package DESeq2 (39). Raw read counts thus obtained were normalized to account for differences in sequencing depth and composition using the median of ratios method implemented within DESeq2. Differential expression of pairwise comparisons (of the different groups) was assessed using the negative binomial test with Benjamini–Hochberg false discovery rate (FDR) adjustment (40) applied for multiple testing corrections. For this study, an FDR of 0.05 was applied, and all candidate genes with a log_2_ fold change value more or less than 1 and with a *p*‐adjusted value of ≤ 0.05 were considered to be significantly up‐ or downregulated. Heatmaps were produced with the ClustVis web tool (41) using the transcript counts as input values.

### Data availability.

The whole-genome shotgun projects were deposited in the Sequence Read Archive (SRA) database of the NCBI under the accessions number SRR10273262 and SRR9209608. RNA-seq data were deposited in NCBI's Gene Expression Omnibus (42) and are accessible through GEO Series accession number GSE140753.
