# Supplementary material for: Survival Strategies and Metabolic Interactions between Ruminococcus gauvreauii and Ruminococcoides bili, Isolated from Human Bile
Source: Microbiol Spectr. 2022 Jul 11;10(4):e02776-21. doi: 10.1128/spectrum.02776-21 (PMC9431564; doi:10.1128/spectrum.02776-21)
Supplement: Supplemental file 1 — Supplemental material. Download spectrum.02776-21-s0001.pdf, PDF file, 0.5 MB [file spectrum.02776-21-s0001.pdf]

SUPPLEMENTARY MATERIAL

Supplementary figures

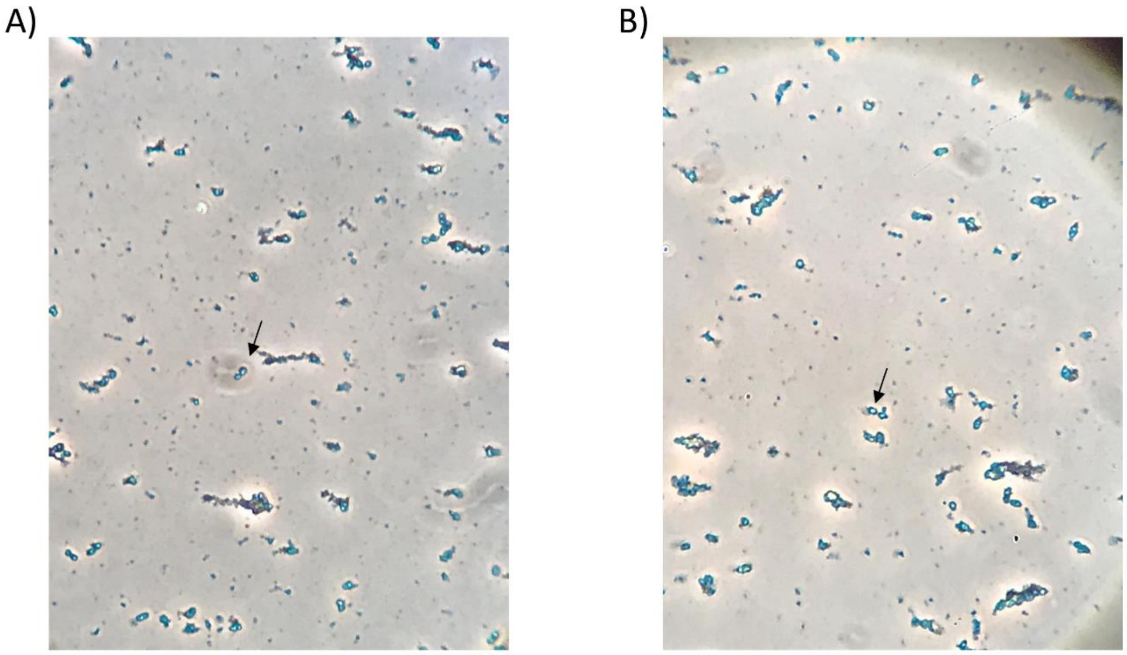

Suppl. Fig1. Microscopy images of spores of *R. gauvreauii* IPLA60001 (A) and DSM-19829 (B) by Schaeffer and Fulton Spore staining.

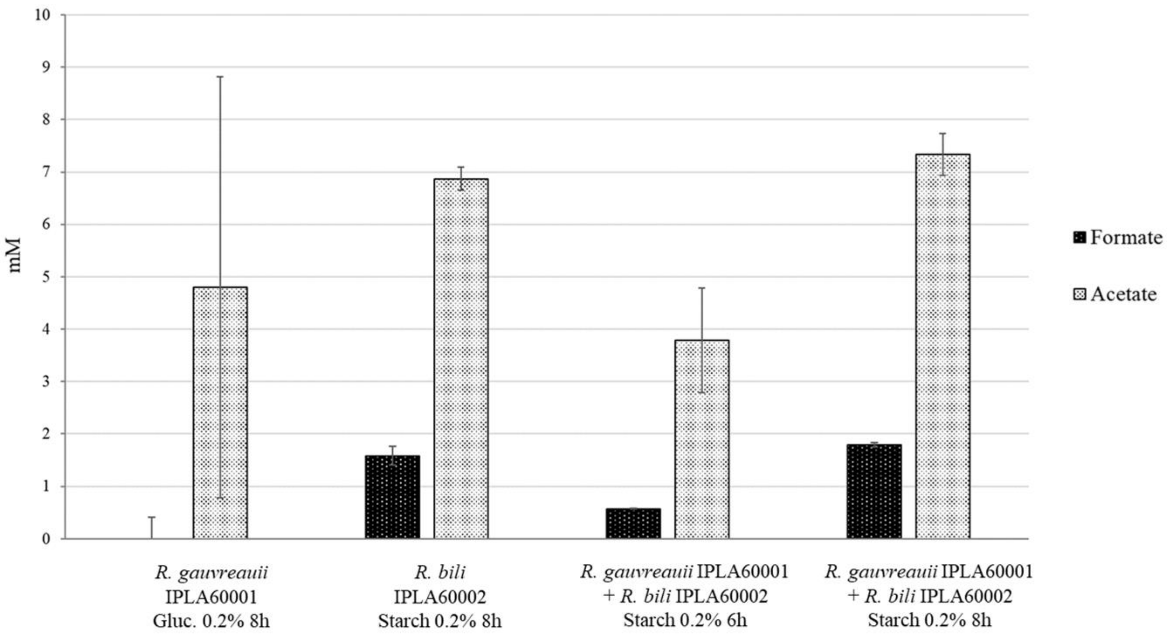

Suppl. Fig. 2. Formate and acetate production of *Ruminococcus gauvreauii* IPLA60001 and *Ruminococcoides bili* IPLA60002 in single cultures during 8 h of growth (IPLA60001 in 0.2% D-glucose and IPLA60002 in 0.2% rice starch, as carbon source) and co-cultures of both strains in rice starch after 6 and 8 h of growth. The results are normalized to blank media controls, and means and standard deviations are represented.

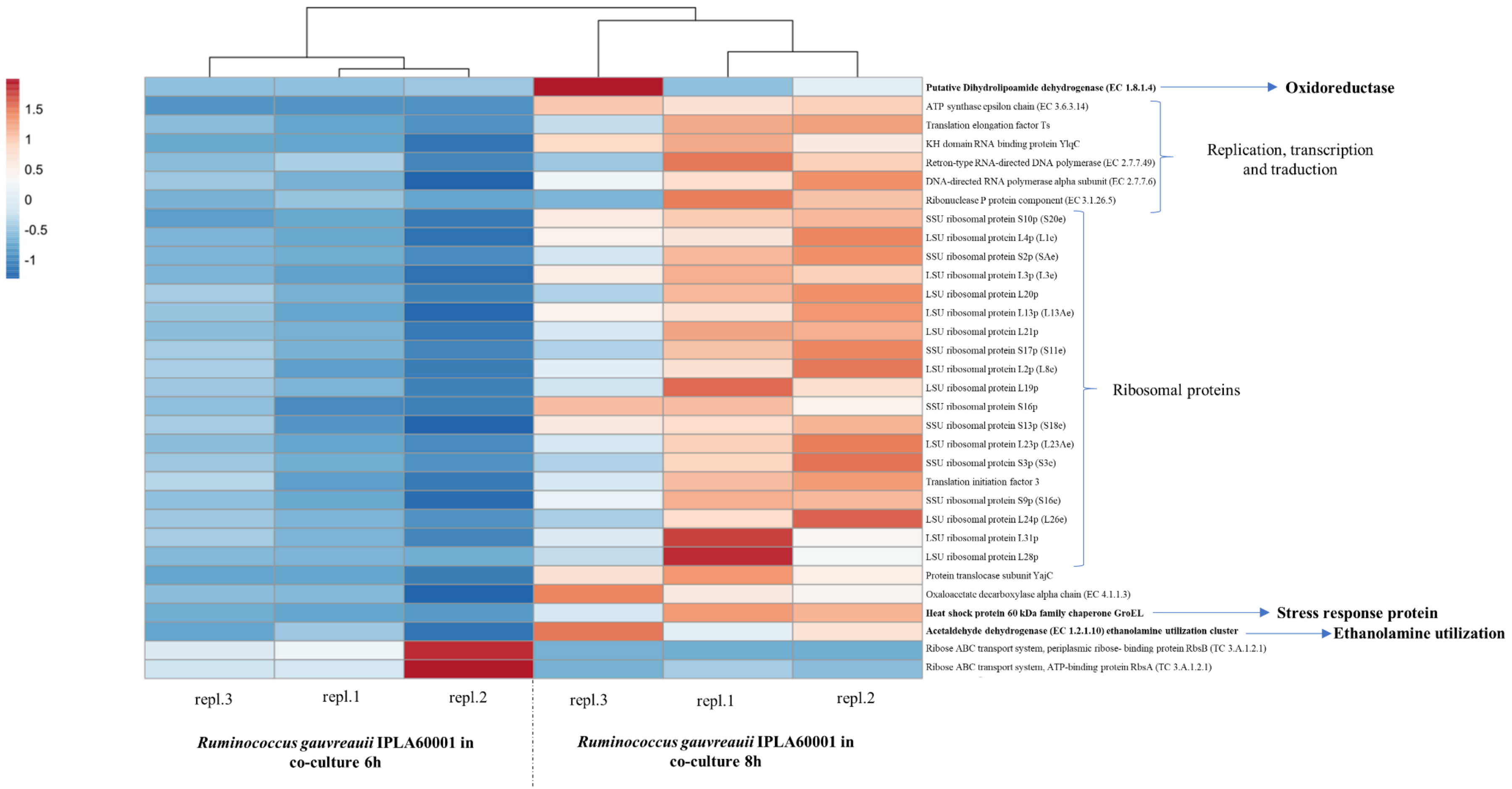

Suppl. Fig. 3. Heatmap of the transcription level of differentially expressed *Ruminococcus gauvreauii* IPLA60001 genes between 6 and 8 h of growth in co-culture with *Ruminococcoides bili* IPLA60002. The heatmap shows only the genes that were differentially expressed with a log2 (fold change) < -1 or > 1 and an adjusted *p*-value of 0.05.

Supplementary Tables

Suppl. Table 1. Differences in genes encoding for vitamin, cofactors and amino acid production pathways between *Ruminococcoides bili* IPLA60002 and *Ruminococcus gauvreauii* IPLA60001. Absence (-) and presence (+) are marked with negative and positive symbols.

Subsystem	Role	IPLA60001	IPLA60002
**Vitamins and cofactors**			
Biotin biosynthesis	Substrate-specific component BioY of biotin ECF transporter	+	+
	Long-chain-fatty-acid--CoA ligase (EC 6.2.1.3)	+	-
	3-ketoacyl-CoA thiolase (EC 2.3.1.16)	+	-
	Biotin operon repressor	+	+
	Adenosylmethionine-8-amino-7-oxononanoate aminotransferase (EC 2.6.1.62)	-	+
	ATPase component BioM of energizing module of biotin ECF transporter	-	+
Biotin biosynthesis Experimental	Competence protein F homolog, phosphoribosyltransferase domain	+	+
	Adenosylmethionine-8-amino-7-oxononanoate aminotransferase (EC 2.6.1.62)	-	+
Thiamine biosynthesis	Sulfur carrier protein adenylyltransferase ThiF	+	+
	tRNA S(4)U 4-thiouridine synthase (former ThiI)	+	-
	Substrate-specific component ThiT of thiamin ECF transporter	+	+
	Thiazole biosynthesis protein ThiG	+	+
	Thiamin pyrophosphokinase (EC 2.7.6.2)	+	+
	Thiamin-phosphate pyrophosphorylase (EC 2.5.1.3)	+	+
	Sulfur carrier protein ThiS	-	+
	1-deoxy-D-xylulose 5-phosphate synthase (EC 2.2.1.7)	+	+
	Hydroxyethylthiazole kinase (EC 2.7.1.50)	+	-
	Hydroxymethylpyrimidine ABC transporter, transmembrane component	+	-
	Predicted hydroxymethylpyrimidine transporter CytX	+	-
	Thiamine-monophosphate kinase (EC 2.7.4.16)	+	-
	Hydroxymethylpyrimidine ABC transporter, ATPase component	+	-
Riboflavin, FMN and FAD metabolism	FMN adenylyltransferase (EC 2.7.7.2)	+	+
	Substrate-specific component RibU of riboflavin ECF transporter	+	+
	Riboflavin kinase (EC 2.7.1.26)	-	+
Riboflavin, FMN and FAD metabolism in plants	FIG000859: hypothetical protein YebC	+	+
	Molybdopterin binding motif, CinA N-terminal domain	+	+
	Multi antimicrobial extrusion protein (Na(+)/drug antiporter), MATE family of MDR efflux pumps	+	+
	C-terminal domain of CinA type S	+	+
	6,7-dimethyl-8-ribityllumazine synthase (EC 2.5.1.78)	+	-
	5-amino-6-(5-phosphoribosylamino)uracil reductase (EC 1.1.1.193)	+	-
	tRNA pseudouridine synthase B (EC 4.2.1.70)	+	+

	Riboflavin kinase (EC 2.7.1.26)	+	-
	GTP cyclohydrolase II (EC 3.5.4.25)	+	-
	Riboflavin synthase eubacterial/eukaryotic (EC 2.5.1.9)	+	-
	Diaminohydroxyphosphoribosylaminopyrimidine deaminase (EC 3.5.4.26)	+	-
	3,4-dihydroxy-2-butanone 4-phosphate synthase (EC 4.1.99.12)	+	-
	FMN adenylyltransferase (EC 2.7.7.2)	-	+
	Substrate-specific component RibU of riboflavin ECF transporter	-	+
	Riboflavin kinase (EC 2.7.1.26)	-	+
Riboflavin to FAD	FMN adenylyltransferase (EC 2.7.7.2)	+	+
	Riboflavin synthase eubacterial/eukaryotic (EC 2.5.1.9)	+	-
	Riboflavin kinase (EC 2.7.1.26)	+	+
	3,4-dihydroxy-2-butanone 4-phosphate synthase (EC 4.1.99.12)	+	-
Pyridoxine (Vitamin B6) Biosynthesis	Pyridoxine biosynthesis glutamine amidotransferase, synthase subunit (EC 2.4.2.-)	-	+
	D-3-phosphoglycerate dehydrogenase (EC 1.1.1.95)	+	+
	Pyridoxamine 5'-phosphate oxidase (EC 1.4.3.5)	+	+
	Pyridoxal kinase (EC 2.7.1.35)	-	+
	Phosphoserine aminotransferase (EC 2.6.1.52)	+	+
	1-deoxy-D-xylulose 5-phosphate synthase (EC 2.2.1.7)	+	+
	Pyridoxine biosynthesis glutamine amidotransferase, glutaminase subunit (EC 2.4.2.-)	-	+
	NAD-dependent glyceraldehyde-3-phosphate dehydrogenase (EC 1.2.1.12)	+	+
	Predicted transcriptional regulator of pyridoxine metabolism	+	-
Folate biosynthesis cluster	Cell division protein FtsH (EC 3.4.24.-)	+	+
	Pantoate--beta-alanine ligase (EC 6.3.2.1)	-	+
	tRNA(Ile)-lysidine synthetase (EC 6.3.4.19)	+	+
	Hypoxanthine-guanine phosphoribosyltransferase (EC 2.4.2.8)	+	+
	GTP cyclohydrolase I (EC 3.5.4.16) type 1	+	-
	Dihydropteroate synthase (EC 2.5.1.15)	+	-
	Aspartate 1-decarboxylase (EC 4.1.1.11)	-	+
	Dihydroneopterin aldolase (EC 4.1.2.25)	+	-
	2-amino-4-hydroxy-6-hydroxymethyldihydropteridine pyrophosphokinase (EC 2.7.6.3)	+	-
Folate Biosynthesis	GTP cyclohydrolase I (EC 3.5.4.16) type 1	+	-
	Dihydrofolate reductase (EC 1.5.1.3)	+	-
	Dihydropteroate synthase (EC 2.5.1.15)	+	-
	Thymidylate synthase (EC 2.1.1.45)	+	-
	Aminodeoxychorismate lyase (EC 4.1.3.38)	+	-
	2-amino-4-hydroxy-6-hydroxymethyldihydropteridine pyrophosphokinase (EC 2.7.6.3)	+	-
	Putative DHNTP pyrophosphatase	+	-
	Dihydroneopterin aldolase (EC 4.1.2.25)	+	-
	Dihydrofolate synthase (EC 6.3.2.12)	+	+
	Folylpolyglutamate synthase (EC 6.3.2.17)	+	+
	Substrate-specific component FolT of folate ECF transporter	-	+
	5-formyltetrahydrofolate cyclo-ligase (EC 6.3.3.2)	+	+
	Thymidylate synthase thyX (EC 2.1.1.-)	-	+
	Para-aminobenzoate synthase, amidotransferase component (EC 2.6.1.85)	+	+
	Aspartate 1-decarboxylase (EC 4.1.1.11)	-	+

Coenzyme A biosynthesis	3-methyl-2-oxobutanoate hydroxymethyltransferase (EC 2.1.2.11)	-	+
	Dephospho-CoA kinase (EC 2.7.1.24)	+	+
	Pantothenate kinase type III, CoaX-like (EC 2.7.1.33)	+	+
	Aspartate 1-decarboxylase (EC 4.1.1.11)	-	+
	Ketol-acid reductoisomerase (EC 1.1.1.86)	+	+
	Phosphopantetheine adenylyltransferase (EC 2.7.7.3)	+	+
	Phosphopantothenoylcysteine synthetase (EC 6.3.2.5)	+	+
	Pantoate--beta-alanine ligase (EC 6.3.2.1)	-	+
	Phosphopantothenoylcysteine decarboxylase (EC 4.1.1.36)	+	+
	Substrate-specific component PanT of predicted pantothenate ECF transporter	+	-
**Amino acids**			
Glutamine, Glutamate, Aspartate and Asparagine biosynthesis	Aspartate--ammonia ligase (EC 6.3.1.1)	+	-
	Aspartate aminotransferase (EC 2.6.1.1)	+	+
	NAD-specific glutamate dehydrogenase (EC 1.4.1.2)	+	-
	Glutamate racemase (EC 5.1.1.3)	+	+
	Asparagine synthetase [glutamine-hydrolyzing] (EC 6.3.5.4)	+	+
	Glutamate synthase [NADPH] large chain (EC 1.4.1.13)	+	+
	Glutamine synthetase type I (EC 6.3.1.2)	+	-
	Glutamate synthase [NADPH] small chain (EC 1.4.1.13)	+	+
	Glutamine synthetase type III, GlnN (EC 6.3.1.2)	+	+
	NADP-specific glutamate dehydrogenase (EC 1.4.1.4)	+	+
	L-asparaginase (EC 3.5.1.1)	-	+
Glutamate dehydrogenases	NADP-specific glutamate dehydrogenase (EC 1.4.1.4)	+	+
	NAD-specific glutamate dehydrogenase (EC 1.4.1.2)	+	-
Glutamine synthetases	Glutamine synthetase type I (EC 6.3.1.2)	+	-
	Glutamine synthetase type III, GlnN (EC 6.3.1.2)	+	+
Polyamine metabolism	ABC transporter, periplasmic spermidine putrescine-binding protein PotD	-	+
	Spermidine Putrescine ABC transporter permease component potC	-	+
	Spermidine Putrescine ABC transporter permease component PotB	-	+
	Ornithine decarboxylase (EC 4.1.1.17)	-	+
	Arginine/ornithine antiporter ArcD	+	-
	Spermidine synthase (EC 2.5.1.16)	+	+
	Carbamate kinase (EC 2.7.2.2)	+	-
	Carboxynorspermidine decarboxylase, putative (EC 4.1.1.-)	+	+
	Putrescine transport ATP-binding protein PotA (TC 3.A.1.11.1)	+	+
	Agmatine deiminase (EC 3.5.3.12)	+	+
	Arginine decarboxylase (EC 4.1.1.19)	+	+
	Carboxynorspermidine dehydrogenase, putative (EC 1.1.1.-)	+	+
	N-carbamoylputrescine amidase (3.5.1.53)	+	+
	Transcriptional regulator, MerR family, near polyamine transporter	+	-
	5'-methylthioadenosine nucleosidase (EC 3.2.2.16)	+	+
Arginine and Ornithine degradation	Arginine/ornithine antiporter ArcD	+	-
	Carbamate kinase (EC 2.7.2.2)	+	-
	Arginine pathway regulatory protein ArgR, repressor of arg regulon	+	+
	Agmatine deiminase (EC 3.5.3.12)	+	+
	Arginine decarboxylase (EC 4.1.1.19)	+	+
	NADP-specific glutamate dehydrogenase (EC 1.4.1.4)	+	+
	Ornithine carbamoyltransferase (EC 2.1.3.3)	+	+

	N-carbamoylputrescine amidase (3.5.1.53)	+	+
	Ornithine decarboxylase (EC 4.1.1.17)	-	+
	Lysine-arginine-ornithine-binding periplasmic protein precursor (TC 3.A.1.3.1)	-	+
Arginine biosynthesis extended	Glutamate N-acetyltransferase (EC 2.3.1.35)	+	-
	Predicted amino-acid acetyltransferase (EC 2.3.1.1)	-	+
	Acetylglutamate kinase (EC 2.7.2.8)	+	+
	Arginine pathway regulatory protein ArgR, repressor of arg regulon	+	+
	Argininosuccinate lyase (EC 4.3.2.1)	+	+
	N-acetylglutamate synthase (EC 2.3.1.1)	+	+
	Argininosuccinate synthase (EC 6.3.4.5)	+	+
	N-acetyl-gamma-glutamyl-phosphate reductase (EC 1.2.1.38)	+	+
	Ornithine carbamoyltransferase (EC 2.1.3.3)	+	+
	Acetylornithine aminotransferase (EC 2.6.1.11)	+	+
Arginine biosynthesis	Glutamate N-acetyltransferase (EC 2.3.1.35)	+	+
	Acetylglutamate kinase (EC 2.7.2.8)	+	+
	Arginine pathway regulatory protein ArgR, repressor of arg regulon	+	+
	Argininosuccinate lyase (EC 4.3.2.1)	+	+
	N-acetylglutamate synthase (EC 2.3.1.1)	+	+
	Argininosuccinate synthase (EC 6.3.4.5)	+	+
	N-acetyl-gamma-glutamyl-phosphate reductase (EC 1.2.1.38)	+	+
	Ornithine carbamoyltransferase (EC 2.1.3.3)	+	+
	Acetylornithine aminotransferase (EC 2.6.1.11)	+	+
	Predicted amino-acid acetyltransferase (EC 2.3.1.1)	-	+
Arginine Deiminase pathway	Arginine/ornithine antiporter ArcD	+	-
	Arginine pathway regulatory protein ArgR, repressor of arg regulon	+	+
	Carbamate kinase (EC 2.7.2.2)	+	-
	Ornithine carbamoyltransferase (EC 2.1.3.3)	+	+
Methionine degradation	S-adenosylhomocysteine nucleosidase (EC 3.2.2.9)	+	+
	Pyruvate-flavodoxin oxidoreductase (EC 1.2.7.-)	+	+
	Methionine ABC transporter ATP-binding protein	+	+
	S-ribosylhomocysteine lyase (EC 4.4.1.21)	+	+
	S-adenosylmethionine synthetase (EC 2.5.1.6)	+	+
	Methionine transporter MetT	+	-
	Methionine gamma-lyase (EC 4.4.1.11)	-	+
Lysine biosynthesis DAP pathway	Diaminopimelate decarboxylase (EC 4.1.1.20)	+	+
	L,L-diaminopimelate aminotransferase (EC 2.6.1.83)	+	+
	Diaminopimelate epimerase (EC 5.1.1.7)	+	-
	Aspartate-semialdehyde dehydrogenase (EC 1.2.1.11)	+	+
	4-hydroxy-tetrahydrodipicolinate reductase (EC 1.17.1.8)	+	+
	Aspartokinase (EC 2.7.2.4)	+	+
	4-hydroxy-tetrahydrodipicolinate synthase (EC 4.3.3.7)	+	+
	Meso-diaminopimelate D-dehydrogenase (EC 1.4.1.16)	+	-
Lysine degradation	Lysine decarboxylase (EC 4.1.1.18)	+	+
	Lysine 2,3-aminomutase (EC 5.4.3.2)	+	-
Threonine degradation	Threonine dehydrogenase and related Zn-dependent dehydrogenases	+	-
	Threonine dehydratase (EC 4.3.1.19)	+	+
	L-threonine 3-dehydrogenase (EC 1.1.1.103)	+	-
Cysteine biosynthesis	Cysteine synthase (EC 2.5.1.47)	+	+
	Serine acetyltransferase (EC 2.3.1.30)	+	+
	Sulfate transport system permease protein CysT	-	+
	Sulfate adenylyltransferase subunit 2 (EC 2.7.7.4)	-	+

	Sulfate adenylyltransferase subunit 1 (EC 2.7.7.4)	-	+
	Sulfate and thiosulfate import ATP-binding protein CysA (EC 3.6.3.25)	-	+
	Sulfate and thiosulfate binding protein CysP	-	+
	Sulfate transport system permease protein CysW	-	+
Common Pathway For Synthesis of Aromatic Compounds (DAHP synthase to chorismate)	5-Enolpyruvylshikimate-3-phosphate synthase (EC 2.5.1.19)	+	+
	2-keto-3-deoxy-D-arabino-heptulosonate-7-phosphate synthase I alpha (EC 2.5.1.54)	+	+
	3-dehydroquinate synthase (EC 4.2.3.4)	+	+
	Chorismate synthase (EC 4.2.3.5)	+	+
	3-dehydroquinate dehydratase I (EC 4.2.1.10)	+	-
	Shikimate kinase I (EC 2.7.1.71)	+	+
	2-keto-3-deoxy-D-arabino-heptulosonate-7-phosphate synthase I beta (EC 2.5.1.54)	+	-
	Shikimate/quinate 5-dehydrogenase I beta (EC 1.1.1.282)	+	-
	Shikimate 5-dehydrogenase I alpha (EC 1.1.1.25)	-	+
	3-dehydroquinate dehydratase II (EC 4.2.1.10)	-	+
Chorismate synthesis	5-Enolpyruvylshikimate-3-phosphate synthase (EC 2.5.1.19)	+	+
	Chorismate mutase I (EC 5.4.99.5)	+	-
	2-keto-3-deoxy-D-arabino-heptulosonate-7-phosphate synthase I alpha (EC 2.5.1.54)	+	+
	Prephenate dehydratase (EC 4.2.1.51)	+	+
	3-dehydroquinate synthase (EC 4.2.3.4)	+	+
	Chorismate synthase (EC 4.2.3.5)	+	+
	Prephenate dehydrogenase (EC 1.3.1.12)	+	-
	3-dehydroquinate dehydratase I (EC 4.2.1.10)	+	-
	Shikimate kinase I (EC 2.7.1.71)	+	+
	2-keto-3-deoxy-D-arabino-heptulosonate-7-phosphate synthase I beta (EC 2.5.1.54)	+	-
	Shikimate/quinate 5-dehydrogenase I beta (EC 1.1.1.282)	+	-
	Shikimate 5-dehydrogenase I alpha (EC 1.1.1.25)	-	+
	Prephenate and/or arogenate dehydrogenase (EC 1.3.1.12)	-	+
	3-dehydroquinate dehydratase II (EC 4.2.1.10)	-	+
Chorismate: Intermediate for synthesis of Tryptophan, PAPA antibiotics, PABA, 3-hydroxyanthranilate and more.	Phosphoribosylformimino-5-aminoimidazole carboxamide ribotide isomerase	+	+
	Anthranilate synthase, amidotransferase component (EC 4.1.3.27)	+	+
	Aminodeoxychorismate lyase (EC 4.1.3.38)	+	-
	Tryptophan synthase alpha chain (EC 4.2.1.20)	+	+
	Anthranilate phosphoribosyltransferase (EC 2.4.2.18)	+	+
	Tryptophan synthase beta chain (EC 4.2.1.20)	+	+
	Indole-3-glycerol phosphate synthase (EC 4.1.1.48)	+	+
	Anthranilate synthase, aminase component (EC 4.1.3.27)	+	+
	Phosphoribosylanthranilate isomerase (EC 5.3.1.24)	+	+
	Para-aminobenzoate synthase, aminase component (EC 2.6.1.85)	+	-
	Tryptophan synthase beta chain like (EC 4.2.1.20)	+	+
	Isochorismatase (EC 3.3.2.1)	+	-
	Para-aminobenzoate synthase, amidotransferase component (EC 2.6.1.85)	+	+
Phenylalanine and Tyrosine branches from Chorismate.	Chorismate mutase I (EC 5.4.99.5)	+	-
	Prephenate dehydratase (EC 4.2.1.51)	+	+
	Prephenate dehydrogenase (EC 1.3.1.12)	+	-
	Biosynthetic Aromatic amino acid aminotransferase alpha (EC 2.6.1.57)	+	-
	Prephenate and/or arogenate dehydrogenase (EC 1.3.1.12)	-	+

Tryptophan synthesis	Anthranilate synthase, amidotransferase component (EC 4.1.3.27)	+	+
	Aminodeoxychorismate lyase (EC 4.1.3.38)	+	-
	Tryptophan synthase alpha chain (EC 4.2.1.20)	+	+
	Para-aminobenzoate synthase, aminase component (EC 2.6.1.85)	+	-
	Anthranilate phosphoribosyltransferase (EC 2.4.2.18)	+	+
	Tryptophan synthase beta chain (EC 4.2.1.20)	+	+
	Indole-3-glycerol phosphate synthase (EC 4.1.1.48)		+
	Para-aminobenzoate synthase, amidotransferase component (EC 2.6.1.85)	+	+
	Anthranilate synthase, aminase component (EC 4.1.3.27)	+	+
	Phosphoribosylanthranilate isomerase (EC 5.3.1.24)	+	+
Glycine Biosynthesis	Serine hydroxymethyltransferase (EC 2.1.2.1)	+	+
	L-threonine 3-dehydrogenase (EC 1.1.1.103)	+	-
Serine Biosynthesis	D-3-phosphoglycerate dehydrogenase (EC 1.1.1.95)	+	+
	Serine hydroxymethyltransferase (EC 2.1.2.1)	+	+
	Phosphoserine aminotransferase (EC 2.6.1.52)	+	+
	Phosphoserine phosphatase (EC 3.1.3.3)	-	+
